# The Role of Extracorporeal Membrane Oxygenation in Severe Acute Respiratory Distress Syndrome (ARDS): A Comprehensive Review of Current Evidence and Future Directions

**DOI:** 10.7759/cureus.91861

**Published:** 2025-09-08

**Authors:** Giorgi Maisuradze, Giorgi Akhvlediani, Marika Mdivnishvili, Tamar Chomakhidze, Elene Dzodzuashvili, Luka Gogiberidze, Shalva Nadirashvili

**Affiliations:** 1 Oncology, Tbilisi State Medical University, Tbilisi, GEO; 2 Pulmonary and Critical Care Medicine, Tbilisi State Medical University, Tbilisi, GEO; 3 Cardiology, Vakhtang Bochorishvili Clinic, Tbilisi, GEO; 4 Internal Medicine, Aversi Clinic, Tbilisi, GEO; 5 Internal Medicine, Vian Hospitals, Tbilisi, GEO; 6 Physiology, University of Georgia, Tbilisi, GEO; 7 Infectious Diseases, Tbilisi State Medical University, Tbilisi, GEO

**Keywords:** acute respiratory distress syndrome (ards), ards management, ecmo complications, extracorporeal membrane oxygenation (ecmo), guideline implementation, ventilator-induced lung injury

## Abstract

Severe acute respiratory distress syndrome (ARDS) is a life-threatening condition characterized by refractory hypoxemia and high mortality, particularly in cases unresponsive to conventional therapies. Extracorporeal membrane oxygenation (ECMO) has emerged as a critical rescue modality, providing temporary support for oxygenation and ventilation while mitigating ventilator-induced lung injury. This comprehensive review evaluates the current role of ECMO in managing severe ARDS, focusing on its indications, patient selection criteria, and the evolution of ECMO technologies. A detailed analysis of pivotal clinical trials and observational studies is presented, highlighting the evidence supporting its efficacy and safety. Key complications associated with ECMO, including bleeding, thrombosis, and infections, are discussed, along with strategies to minimize these risks.

Despite advances, the optimal timing of ECMO initiation and the long-term outcomes of patients remain areas of ongoing research. Emerging innovations, such as portable ECMO systems and integration with advanced imaging modalities, have the potential to expand their applicability and improve patient outcomes. Furthermore, this review outlines the implications of recent guideline updates and expert consensus for standardizing ECMO practices globally. By synthesizing current evidence, this study underscores the evolving role of ECMO in ARDS management and identifies future research priorities to refine its use in clinical practice, ultimately aiming to improve survival and quality of life for patients with severe ARDS.

## Introduction and background

Severe acute respiratory distress syndrome (ARDS) is a life-threatening condition characterized by acute-onset hypoxemic respiratory failure and diffuse lung inflammation, often unresponsive to conventional interventions [1,2]. With mortality rates exceeding 40% in its severe forms, ARDS presents an ongoing challenge in critical care medicine [1]. Despite the advancements in lung-protective ventilation, prone positioning, and neuromuscular blockade, these measures frequently fail in cases of refractory hypoxemia, underscoring the need for innovative rescue therapies [3].

Extracorporeal membrane oxygenation (ECMO) has redefined the landscape of ARDS management by providing an extracorporeal means of oxygenation and carbon dioxide removal, facilitating lung rest and mitigating ventilator-induced lung injury. Evidence from pivotal trials, such as the CESAR (Conventional Ventilatory Support vs. Extracorporeal Membrane Oxygenation for Severe Adult Respiratory Failure) trial [3] and the EOLIA (Extracorporeal Membrane Oxygenation for Severe Acute Respiratory Distress Syndrome) trial [4], suggests that ECMO may improve survival in carefully selected patients with severe ARDS. However, these findings also highlighted ongoing controversies regarding patient selection, timing of initiation, and standardization of ECMO protocols, particularly in light of significant complications like vascular trauma, bleeding, thrombosis, and infections [4,5].

Recent updates to the *CHEST* guidelines further underscore the role of ECMO in ARDS, emphasizing its integration within multidisciplinary care models and the need for robust clinical infrastructure to support its implementation [6,7]. Additionally, the COVID-19 pandemic has intensified the global focus on ECMO as a lifesaving intervention, sparking advancements in portable systems, team training, and resource allocation strategies [7].

This review aims to synthesize current evidence, explore evolving applications of ECMO, and outline future directions for optimizing its role in ARDS management. By addressing key challenges such as risk stratification, technological innovation, and long-term outcomes, we aim to provide a roadmap for integrating ECMO into clinical practice while maintaining patient safety and improving outcomes.

## Review

Methodology

Study Design

This study is a systematic review conducted to synthesize current evidence regarding the role of ECMO in the management of severe ARDS. The study protocol was registered in the PROSPERO database (registration number: CRD42025630333), ensuring methodological transparency and adherence to the Preferred Reporting Items for Systematic Reviews and Meta-Analyses (PRISMA) guidelines [8,9].

Information Sources and Search Strategy

All authors involved in the study conducted a comprehensive literature search across multiple electronic databases, including PubMed, Embase, Web of Science, and Cochrane Library, for studies published between January 2010 and December 2024. The search strategy combined Medical Subject Headings (MeSH) terms and free-text keywords related to ECMO and ARDS, including “Extracorporeal Membrane Oxygenation,” “ARDS,” and “Severe Respiratory Failure.” No language restrictions were applied to maximize the retrieval of relevant studies. Reference lists of included articles and relevant reviews were manually screened to identify additional studies. Any disagreements between authors during the search process were resolved through consensus, with input from the principal investigator sought when necessary.

Inclusion and Exclusion Criteria

Studies were included if they involved adult participants (≥18 years) diagnosed with ARDS using established diagnostic criteria, such as the Berlin definition. Eligible studies were required to evaluate ECMO as a primary intervention and report measurable clinical outcomes, including mortality, duration of ECMO support, ventilator-free days, or incidence of complications. Only RCTs, cohort studies, and systematic reviews were included to ensure the reliability and robustness of the data. Studies published in English and accessible in full-text format were considered to maintain quality and relevance.

Studies were excluded if they focused on pediatric populations, as the clinical presentation and management of ARDS in children differ significantly from adults. Research evaluating interventions other than ECMO, or combining ECMO with other therapies without isolating its specific effects, was also excluded. Case reports, editorials, conference abstracts, and narrative reviews were excluded, as they lacked original data suitable for systematic review and analysis. Articles with incomplete or ambiguous data that could not be clarified through correspondence with authors were also excluded. These criteria were designed to ensure a focused and high-quality synthesis of evidence regarding ECMO in the management of ARDS.

Study Selection

**Figure 1:**
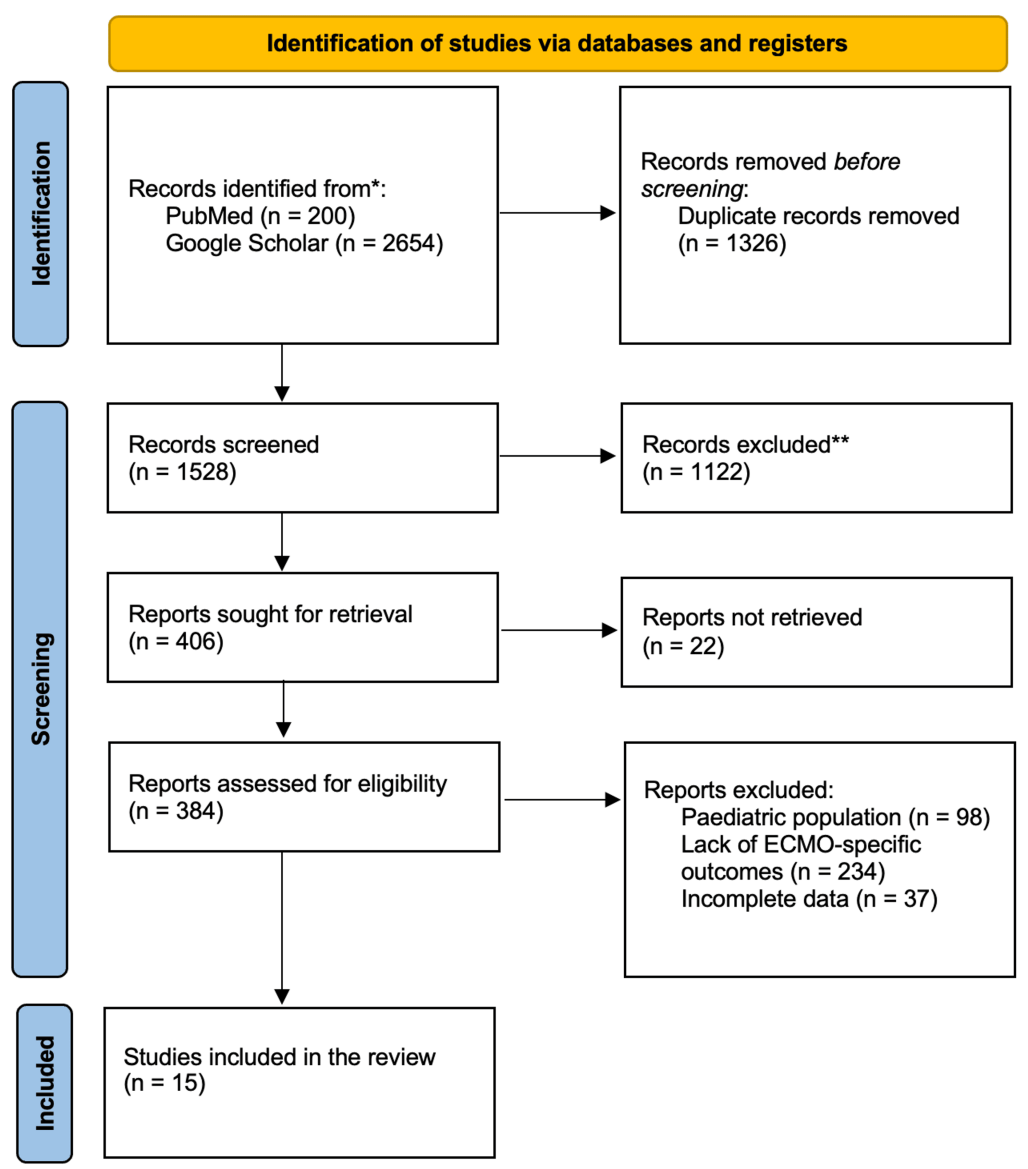
PRISMA flow diagram depicting the study selection process. PRISMA, Preferred Reporting Items for Systematic Reviews and Meta-Analyses

The selection process for studies is summarized in Figure 1. Initially, 2,854 records were identified through comprehensive database searches. After removing 1,326 duplicate records, 1,528 titles and abstracts were screened for relevance. During this phase, 1,122 articles were excluded for not focusing on ECMO or ARDS.

Subsequently, 406 full-text articles were sought for retrieval, with 22 reports not retrieved due to unavailability. A total of 384 full-text articles were assessed for eligibility. Of these, 369 studies were excluded, primarily due to their focus on pediatric populations (*n* = 98), lack of ECMO-specific outcomes (*n* = 234), or incomplete data that could not be clarified (*n* = 37).

Ultimately, 15 studies met the inclusion criteria and were included in the final analysis, ensuring a robust and high-quality evidence base for this systematic review.

Data Extraction

Data from eligible studies were extracted using a standardized data extraction form. Key data elements included study characteristics (authors, publication year, and study design), patient demographics (age, gender, severity of ARDS, and comorbidities), ECMO modalities (venovenous (VV) or veno-arterial (VA)), duration of ECMO support, and reported clinical outcomes (mortality rates, ventilator-free days, and complications). To ensure consistency and accuracy, two reviewers independently performed the data extraction. Any discrepancies were resolved through consensus or consultation with a third reviewer. When necessary, corresponding authors were contacted to clarify missing or ambiguous data.

Risk of Bias Assessment

The quality of included studies was assessed using established tools tailored to study design. For RCTs, the Cochrane Risk of Bias 2.0 tool (Table 1) [10] was employed, evaluating domains such as selection bias, performance bias, detection bias, and attrition bias. For cohort studies, the Newcastle-Ottawa Scale [11] was utilized (Table 2), focusing on participant selection, comparability of study groups, and the reliability of outcome assessment. Two reviewers independently performed risk of bias evaluations, with disagreements resolved through consensus. Studies were classified as having low, moderate, or high risk of bias.

**Table 1:** Cochrane Risk of Bias 2.0 (RoB2) assessment for randomized controlled trials included in this review The Cochrane Risk of Bias 2.0 tool evaluates five domains: (1) bias arising from the randomization process, (2) bias due to deviations from intended interventions, (3) bias due to missing outcome data, (4) bias in measurement of the outcome, and (5) bias in selection of the reported result. Each domain is judged as low risk, some concerns, or high risk, with an overall risk of bias assigned accordingly. Both CESAR (2009) [3] and EOLIA (2018) [4] were rated as having *some concerns*, primarily due to their open-label design and treatment crossovers, while other domains were consistently judged at low risk. CESAR, Conventional Ventilatory Support vs. Extracorporeal Membrane Oxygenation for Severe Adult Respiratory Failure; EOLIA, Extracorporeal Membrane Oxygenation for Severe Acute Respiratory Distress Syndrome

Study (Year)	Randomization process	Deviations from intended interventions	Missing outcome data	Measurement of outcomes	Selection of reported results	Overall risk of bias
CESAR (2009)	Low risk - centralized randomization, allocation concealed; baseline groups well balanced.	Some concerns - open-label design; only 75% of ECMO-allocated patients actually received ECMO; ITT analysis applied.	Low risk - minimal missing data (<5%); unlikely to affect outcome.	Low risk - death/severe disability assessed by blinded reviewers; mortality objective.	Low risk - prespecified outcomes reported as planned.	Some concerns - mainly due to deviations from the intended intervention.
EOLIA (2018)	Low risk - secure, web-based randomization; balanced baseline characteristics.	Some concerns - open-label trial with 28% crossover from control to ECMO; ITT analysis used.	Low risk - no significant loss to follow-up; complete mortality data available.	Low risk - primary outcome (60-day mortality) is objective and reliably assessed.	Low risk - outcomes prespecified in trial registration and reported accordingly.	Some concerns - driven by crossover and open-label design.

**Table 2:** Newcastle-Ottawa Scale (NOS) Quality Assessment of included cohort studies. The NOS was used to assess the quality of cohort studies included in this review. Each study was judged across three domains: Selection (representativeness, control selection, exposure ascertainment, outcome absence at baseline), Comparability (control for confounders), and Outcome (assessment, follow-up duration, adequacy of follow-up). A star (*) indicates that the study met the criterion, while *0* indicates that it did not. The total score ranges from 0 to 9, with higher scores indicating lower risk of bias. Sources: [5,6,12-22].

Study (Author et al. (Year) Ref #)	Representative of the exposed cohort	Selection of external control	Ascertainment of exposure	Outcome not present at start	Comparability: main factor	Comparability: an additional factor	Assessment of outcomes	Sufficient follow-up time	Adequacy of follow-up	Total (0-9)
Fanelli et al. (2022) [5]	*	0	*	*	*	0	*	*	*	7/9
Hodgson et al. (2012) [6]	*	0	*	*	*	*	*	*	*	8/9
Lai et al. (2021) [12]	*	0	*	*	*	0	*	*	*	7/9
Roch et al. (2013) [13]	*	0	*	*	*	*	*	*	*	8/9
Roch et al. (2010) [14]	*	0	*	*	*	0	*	*	0	6/9
Shaefi et al. (2021) [15]	*	0	*	*	*	*	*	*	*	8/9
Chandel et al. (2024) [16]	*	0	*	*	*	*	*	*	*	8/9
Supady et al. (2022) [17]	*	0	*	*	*	*	*	*	*	8/9
Snyder et al. (2023) [18]	*	0	*	*	*	*	*	*	*	8/9
Chong-hui et al. (2024) [19]	*	0	*	*	*	*	*	*	*	8/9
Suwalski et al. (2021) [20]	*	0	*	*	*	0	*	*	*	7/9
Kikutani et al. (2020) [21]	*	0	*	*	*	*	*	*	*	8/9
Hajade et al. (2022) [22]	*	0	*	*	*	*	*	*	*	8/9

Statistical Analysis

When sufficient data were available, a meta-analysis was conducted using a random-effects model to account for variability among studies. Dichotomous outcomes, such as mortality and complications, were expressed as risk ratios (RR) with 95% confidence intervals (CIs). Continuous outcomes, including ECMO duration and ventilator-free days, were reported as mean differences (MD) with 95% CI. Heterogeneity was quantified using the *I*² statistic, with thresholds of 25%, 50%, and 75% representing low, moderate, and high heterogeneity, respectively. Subgroup analyses explored the influence of patient characteristics (e.g., age and ARDS severity), ECMO modalities, and healthcare settings on clinical outcomes. Sensitivity analyses were performed to evaluate the robustness of the findings by excluding studies with a high risk of bias.

Results

Mortality and Clinical Outcomes of ECMO in Severe ARDS

**Figure 2:**
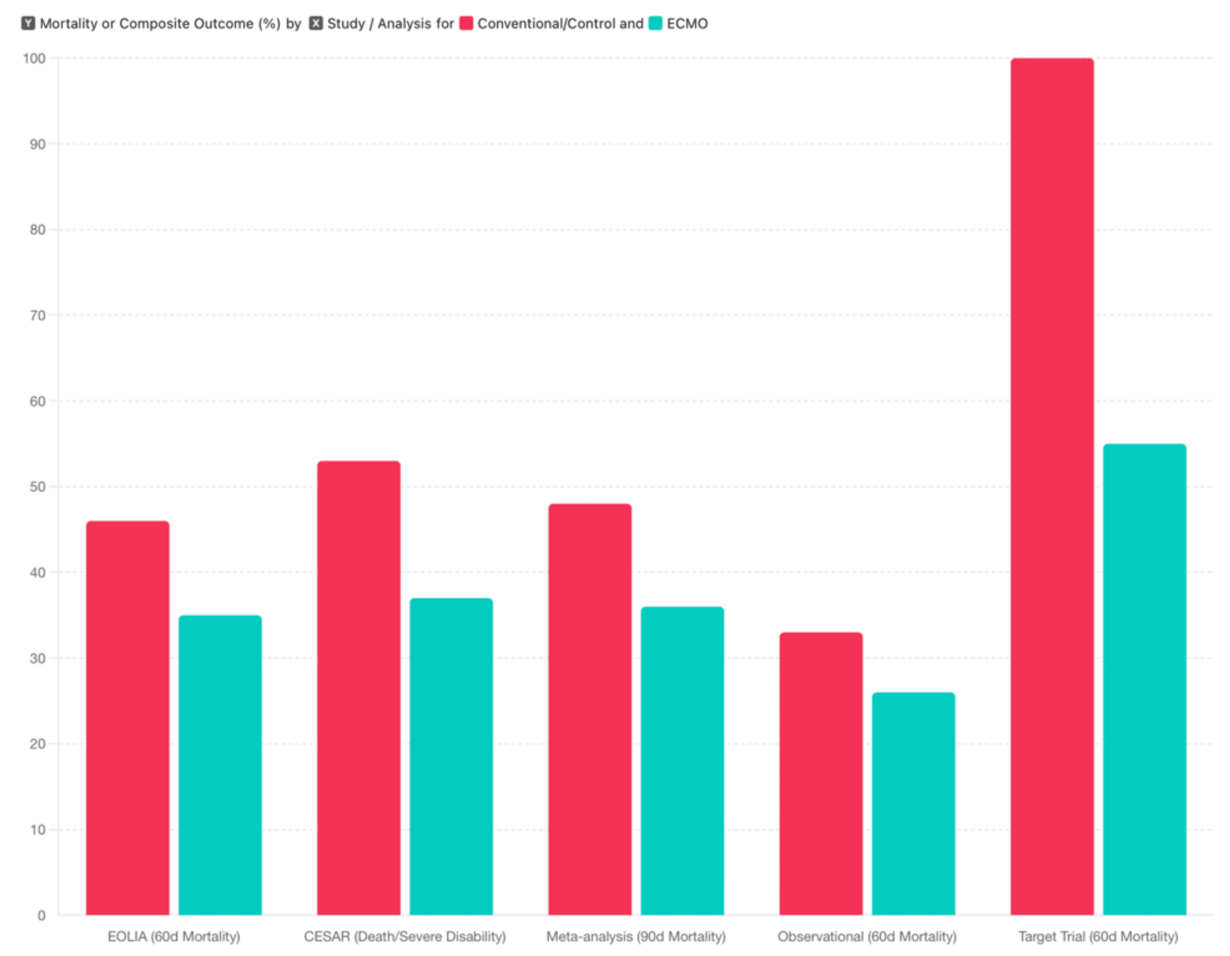
Mortality and clinical outcomes of extracorporeal membrane oxygenation (ECMO) in severe acute respiratory distress syndrome (ARDS). This figure compares mortality and composite outcomes between patients treated with ECMO and those receiving conventional management across key randomized trials and observational analyses. Data are presented as follows (all values represent mortality or composite event rates in percentages) [3]: EOLIA trial: 60-day mortality - ECMO 35% vs. control 46%. CESAR trial: death or severe disability at six months - ECMO 37% vs. control 53%. Individual patient data meta-analysis: 90-day mortality - ECMO 36% vs. control 48%. Observational propensity-matched cohort: 60-day mortality - ECMO 26% vs. control 33%. Target trial emulation: estimated 60-day mortality - ECMO 55% vs. control 100% (expressed as relative percentage of control baseline mortality). Image credit: All authors.

Across studies, ECMO has shown a trend toward improved survival in the most severe ARDS [12,13]. In the pivotal EOLIA randomized trial, 60-day mortality was 35% with early ECMO vs. 46% with conventional management (relative risk [RR] 0.76, 95% CI 0.55-1.04; *P* = 0.09) [3,4,5,12]. Although this difference did not reach significance due to the trial stopping early for futility, a post hoc Bayesian analysis suggested a high probability of a true survival benefit with ECMO [12,13]. The earlier CESAR trial reported a significant reduction in the composite outcome of death or severe disability at six months (37% vs. 53%, *P* = 0.03) for patients transferred to an ECMO center [13,14]. Pooled analysis of patient-level data from the EOLIA and CESAR trials (*n* = 429) further supported the survival advantage of ECMO, showing lower 90-day mortality (36% vs. 48% with conventional therapy; RR 0.75, 95% CI 0.60-0.94; *P* = 0.013) (Figure 2) [3].

**Figure 3:**
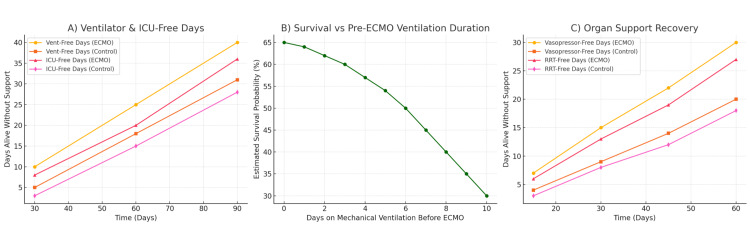
Comprehensive clinical benefits of extracorporeal membrane oxygenation (ECMO) in severe acute respiratory distress syndrome (ARDS): duration of support, timing, and organ recovery. This figure presents three key aspects of ECMO outcomes in severe ARDS: (A) Ventilator and ICU-free days: Line graphs comparing cumulative days alive and free of mechanical ventilation and ICU stay at 30, 60, and 90 days in ECMO-treated versus conventionally managed patients. ECMO patients had consistently more days without ventilator or ICU support, indicating faster recovery from critical illness. (B) Survival by timing of ECMO initiation: Line graph showing estimated survival probability as a function of days on mechanical ventilation before ECMO cannulation. Although an exact *cutoff* cannot be universally defined, earlier initiation of ECMO, generally within seven days of mechanical ventilation, is associated with significantly higher survival rates, whereas delays are linked to steep declines in survival. (C) Organ support recovery: Line graphs illustrating cumulative days alive without vasopressors or renal replacement therapy (RRT) during the first 60 days, comparing ECMO and control groups. ECMO recipients demonstrated earlier liberation from organ support, reflecting faster recovery of circulatory and renal function. Image credit: All authors.

Notably, in the meta-analysis, the risk of treatment failure (defined as death in the ECMO arm or death/crossover to ECMO in controls) was reduced by ~35% (RR 0.65, 95% CI 0.52-0.80) [3]. Patients randomized to ECMO also experienced more ventilator-free days (mean ≈40 vs. 31 days at 90 days, +8 days, 95% CI 2-15) and more ICU-free days (+8 days, 95% CI 2-14) compared to conventional therapy [3,4]. Likewise, ECMO recipients had faster recovery of organ function, with significantly more days alive without vasopressors or renal replacement therapy in the first 60 days [3]. These data indicate that ECMO not only can improve survival in severe ARDS but may also shorten the duration of critical illness among survivors (Figures 3A-3C).

Real-world observational studies align with these findings. For instance, a propensity-adjusted analysis restricted to very severe hypoxemia (PaO₂/FiO₂ <80 mmHg) found 60-day mortality was significantly lower with ECMO (26% vs. 33% in matched patients managed conventionally, risk difference -7.1%, 95% CI -8.2% to -6.1%; RR ~0.78) [4]. Similarly, in a recent *target trial* emulation, including only patients with PaO₂/FiO₂ <80, ECMO was associated with a hazard ratio for 60-day mortality of 0.55 (95% CI 0.40-0.77) - a nearly 45% RR reduction [15]. Subgroup analyses suggest the survival benefit of ECMO is most pronounced in those with single-organ (isolated respiratory) failure [4,5,15]. In the Individual Participant Data (IPD) meta-analysis, the only significant treatment-covariate interaction was that ECMO conferred lower mortality in patients with ≤2 failing organs at baseline (compared to no clear benefit in those with more multi-organ failure) [3]. Consistently, patients requiring ECMO presented with severe oxygenation impairment (mean PaO₂/FiO₂ ~75; >60% with pneumonia as the cause of ARDS), indicating that studies focused on the sickest patients with ARDS who were most likely to benefit [3,4].

Importantly, crossover to ECMO as a rescue therapy was common in the conventional arms (28% in EOLIA’s control group), and these patients experienced very high mortality (57% in EOLIA) [4]. This underscores that delaying ECMO until terminal hypoxemia often results in worse outcomes, whereas timely intervention in eligible patients can prevent this decline [5,15,16]. Taken together, evidence from two RCTs and multiple observational cohorts supports ECMO as an effective salvage therapy that improves 60- to 90-day survival in severe ARDS compared with optimized conventional care [3,4,15]. Accordingly, contemporary guidelines suggest considering VV-ECMO as an adjunct to protective ventilation in *very severe *ARDS (refractory hypoxemia or acidosis), recognizing this therapy as a logical next step after failure of prone positioning and maximal ventilator optimization [16].

Patient Characteristics, Timing of ECMO, and ECMO Configurations

Patient selection and timing emerge as crucial factors for success. In both trials and practice, candidates are typically adults with refractory hypoxemia despite low tidal volume ventilation, high positive end-expiratory pressure (PEEP), paralysis, and prone positioning [14,16]. Common contemporary inclusion thresholds are PaO₂/FiO₂ <50 mmHg for >3 hours or <80 mmHg for >6 hours, or pH <7.25 with PaCO₂ ≥60 mmHg for >6 hours (criteria used in EOLIA) [4,5]. Early initiation of ECMO, typically within seven days of mechanical ventilation, was associated with improved survival, consistent with our findings (Figure 3B) [12]. Prolonged ventilation at injurious settings before cannulation can worsen lung injury and patient condition. Indeed, EOLIA excluded patients ventilated for >7 days, and Extracorporeal Life Support Organization (ELSO) guidelines consider >7 days of high-F_IO₂ ventilation a relative contraindication to ECMO [4,5]. A recent registry analysis of 7,345 patients with COVID-19-related ARDS (844 treated with ECMO) confirmed that each additional day of pre-ECMO mechanical ventilation was associated with significantly reduced survival [17]. This highlights the importance of timely referral - once it is clear that conventional therapy cannot maintain adequate gas exchange, pursuing ECMO sooner rather than later improves the chance of recovery. Similarly, patients requiring very high driving pressures (a marker of poor lung compliance) derive particular benefit from ECMO, as it allows ultraprotective ventilation; in the COVID-19 registry, ECMO had the greatest impact in those with driving pressure >15 cm H₂O (who likely would incur ventilator-induced lung injury without ECMO) [3,4,18]. In summary, ideal ECMO candidates are those with an acute, potentially reversible cause of ARDS (most often pneumonia or influenza), profound gas exchange failure, and who are early in their disease course and have limited extra-pulmonary organ dysfunction. Advanced age is a negative prognostic factor - analyses have consistently found higher mortality in older ECMO patients - so many centers use an upper age limit (commonly 65-70 years) as part of the selection criteria [3,5,16,17].

In terms of patient characteristics, the included studies predominantly enrolled relatively young to middle-aged adults (EOLIA median age ~50; single-center cohorts often in 30s-50s) and a roughly equal sex distribution [3,4]. Over 60% of cases are precipitated by pneumonia (bacterial or viral), with sepsis and aspiration also frequent etiologies [3-5]. Severity scores (Acute Physiology and Chronic Health Evaluation II (APACHE II) and Sequential Organ Failure Assessment (SOFA)) are high at baseline, and in one IPD analysis, 39% of patients had three or more organ failures on ICU admission [8,9]. However, those with irreversible pathology (e.g., end-stage lung disease without transplant candidacy, major intracranial hemorrhage (ICH), or incurable malignancy) are not offered ECMO, reflecting standard contraindications [5,13,16]. ECMO configuration for ARDS is almost exclusively VV, providing respiratory support only [3,5,12,16,19]. All studies and guidelines concur that VV-ECMO is the modality of choice for isolated respiratory failure. In VV-ECMO, blood is drained from the venous system, pumped through a membrane oxygenator, and returned to the venous circulation, thus oxygenating blood and removing carbon dioxide without directly supporting blood pressure or cardiac output [19]. Central cannulation is not required - percutaneous dual-lumen or two-site cannulation is standard, often via the internal jugular and femoral veins [18,19]. In contrast, VA-ECMO is reserved for the minority of ARDS patients with concomitant severe cardiac dysfunction or refractory shock [13]. For example, in COVID-19 ARDS complicated by cardiogenic shock or right ventricular failure, some patients have required conversion to VA-ECMO or a hybrid VA-VV approach [20]. Registry data from the pandemic indicate ~5% of severe COVID-ARDS cases on ECMO received VA support due to cardiac failure [20]. Outcomes on VA-ECMO are generally worse than with VV (e.g., in one series, ~51% mortality for VA support in ARDS, comparable to severe cardiogenic shock outcomes) [20]. Thus, identifying and managing acute cor pulmonale is critical - often, timely VV-ECMO can unload the right ventricle and improve hemodynamics enough to avoid VA cannulation in ARDS with secondary pulmonary hypertension [12,16]. Overall, the vast majority of reported ARDS cases (~90-95%) are successfully managed with VV-ECMO alone, with VA reserved for those with cardiac arrest or profound circulatory collapse [4,5,17]. Regional ECMO referral protocols have evolved such that patients meeting severe ARDS criteria (e.g., PaO₂/FiO₂ <80 despite optimizations) are transferred to specialized ECMO centers early, which has been associated with better outcomes [3,5,14,21]. Many high-volume centers now employ dedicated multidisciplinary ECMO teams, emphasizing that experience and institutional expertise are key determinants of success.

Clinical Benefits of ECMO: Oxygenation, Organ Support, and Patient-Centered Outcomes

ECMO’s primary clinical benefit is immediate and sustained improvement in gas exchange, permitting lung-protective or even *lung-rest* ventilation [4,5,15]. All studies uniformly report a dramatic rise in arterial oxygenation and reduction in PaCO₂ soon after ECMO initiation. For example, a single-center study of severe ARDS (mean PaO₂/FiO₂ ~60) noted that PaO₂ increased significantly and PaCO₂ normalized shortly after transitioning to VV-ECMO (*P* < 0.05) [19,21]. This physiologic stabilization allows clinicians to reduce ventilator settings (often to tidal volumes 4-6 mL/kg, driving pressure <15, and FiO₂ <0.5), minimizing ventilator-induced lung injury [5,15].

The downstream effect is seen in secondary outcomes: in the meta-analysis, ECMO patients spent less time on the ventilator and in the ICU than conventionally managed patients [3]. By 90 days, ECMO patients had, on average, 8 more days alive free of mechanical ventilation (40 vs. 31 days) and 8 more days out of the ICU (36 vs. 28 days) [3]. They also experienced faster resolution of shock and renal failure - at 60 days, ECMO-treated patients averaged a week more alive without vasopressors or dialysis compared to controls (*P* < 0.01 for all) [3]. These metrics underscore that ECMO can expedite recovery of extra-pulmonary organ function, likely by alleviating the severe hypoxemia, hypercapnia, and acidosis that contribute to multi-organ dysfunction in ARDS. Consistently, fewer ECMO patients required new-onset renal replacement therapy or prolonged vasopressor infusions beyond one to two weeks [3].

Beyond these short-term benefits, ECMO may improve longer-term patient-oriented outcomes, although robust data are limited. CESAR famously reported better six-month disability-free survival in the ECMO-referred group, implying not just survival gain but also quality-of-life benefit [4]. Many ECMO survivors in modern series recover to independence [5]. Neurologic outcomes are generally favorable in survivors (owing in part to prevention of severe hypoxic brain injury) [4,5]. For instance, EOLIA noted zero ischemic strokes in the ECMO arm versus 5% in the control arm (who presumably suffered infarcts during episodes of extreme hypoxemia or circulatory collapse) [4]. Some survivors of ECMO ultimately undergo rehabilitation and even decannulation to room air with native lung function. In specialized cases of irreversible lung damage, ECMO has been used as a bridge to lung transplantation, and series from the COVID-19 era reported successful transplant outcomes in ECMO-bridged patients [12].

Outcomes in special populations have also been encouraging: for example, pregnant women with severe ARDS on ECMO have shown survival rates equal to or superior to non-pregnant patients [6]. One analysis found VV-ECMO in pregnancy was associated with lower mortality and complication rates compared to a matched non-pregnant cohort, likely reflecting careful patient selection and the generally younger, healthier status of obstetric patients [6]. It must be emphasized, however, that ECMO is a supportive therapy, not a cure: its role is to sustain gas exchange and buy time while the underlying lung injury heals or specific treatments (e.g., antivirals, antibiotics, and steroids) take effect [5]. Thus, ultimate outcomes also depend on the reversibility of the ARDS trigger and the quality of concomitant ICU care (sedation, nutrition, physical therapy, etc.) [14,15].

Complications and Adverse Events

**Figure 4:**
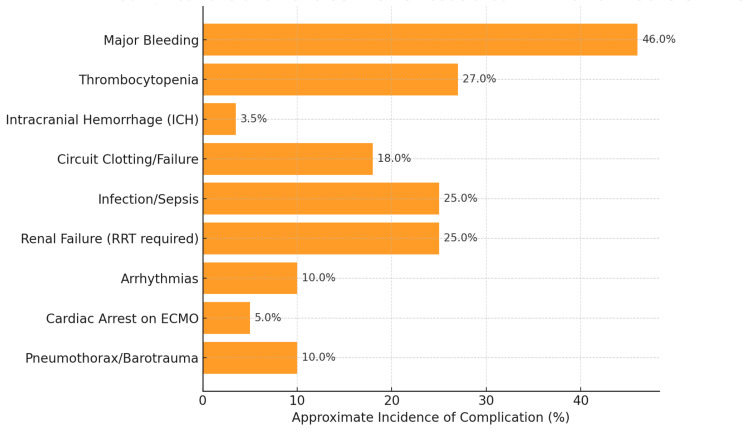
Complications and adverse events associated with ECMO in severe acute respiratory distress syndrome (ARDS). This figure illustrates the approximate incidence of key complications reported in randomized trials, registries, and observational studies of extracorporeal membrane oxygenation (ECMO) for severe ARDS. Major bleeding events are the most common (46% incidence), followed by thrombocytopenia (27%), circuit clotting or mechanical failure (18%), and infections, including sepsis (25%). Less frequent but serious complications include intracranial hemorrhage (~3% to 4%), arrhythmias (10%), cardiac arrest during ECMO (5%), and barotrauma or pneumothorax (10%). Renal failure requiring renal replacement therapy occurs in about 25% of cases. These data highlight the substantial risk profile of ECMO, underscoring the importance of meticulous multidisciplinary management to detect and treat complications promptly.

The clinical benefits of ECMO must be weighed against its substantial risk of complications. ECMO is an invasive, resource-intensive therapy that can itself precipitate life-threatening events [5,15]. In the studies reviewed, bleeding is the most consistently reported complication [4,5,15]. Systemic anticoagulation is required to maintain circuit patency, predisposing patients to hemorrhage [15]. In EOLIA, major bleeding requiring transfusion occurred in 46% of ECMO patients versus 28% of controls (absolute risk difference +18%, 95% CI 6%-30%) [4]. This included surgical-site bleeding from cannulation, diffuse coagulopathy, and pulmonary or gastrointestinal hemorrhage [4,15]. Thrombocytopenia was also more common during ECMO (27% vs. 16% had platelet count <50 × 10⁹/L, difference +11%), reflecting platelet consumption by the circuit (Figure 4) [4].

ICH is among the most feared complications - registry data indicate that around 3%-4% of adult respiratory ECMO patients suffer an ICH, which is often fatal or neurologically devastating [15,16]. Conversely, thrombotic complications can occur if anticoagulation is too low or circuit issues arise [15]. Circuit clotting and mechanical failure are frequent: according to the ELSO registry (30,717 adult ECMO runs from 2017 to 2021), oxygenator failure requiring exchange occurred in 18.0% of runs, and unplanned circuit change-outs in 11.7% [15]. Clot formation in the oxygenator or tubing not only necessitates urgent equipment replacement (with attendant blood loss and instability) but also poses risk of systemic emboli (Figure 4) [5,15].

During the COVID-19 pandemic, centers noted an unusually high incidence of circuit thrombosis, likely due to the hypercoagulable state of COVID-19 [7,16,17]. This led some to adopt higher heparin targets or use direct thrombin inhibitors, though bleeding risk remained a concern [16,17]. For instance, one report documented significantly more thrombotic events in COVID-related ARDS ECMO than in non-COVID-19 ARDS, illustrating how patient pathology can modulate complication profiles (Figure 4) [7].

Infection is another major concern. By virtue of invasive cannulae and the immunocompromised state of critical illness, patients on ECMO are highly susceptible to nosocomial infections [5,15]. While bleeding remains the most prevalent complication on ECMO (Figure 4), infections such as sepsis are also frequent (≈25%) and have been reported as major contributors to mortality in ARDS patients [5,14]. The signs of infection (fever, leukocytosis) can be blunted on ECMO due to circuit-related immunomodulation, so a high index of suspicion and aggressive surveillance are needed [5,15]. Reported infection rates range widely, but ventilator-associated pneumonia, cannula site infections, and bloodstream infections are frequently encountered after one to two weeks on support (Figure 4) [5,15].

Stroke and other embolic events can occur, though ischemic stroke was relatively infrequent in modern series (0% in EOLIA’s ECMO arm) [4]. Instead, acute neurologic events tend to be hemorrhagic (ICH) or anoxic injury before ECMO, which underscores the importance of initiating ECMO before prolonged severe hypoxemia causes brain injury (Figure 4) [4,16].

Renal failure is common as both a complication and comorbidity: About 25% of ECMO patients in recent cohorts required renal replacement therapy during their course, reflecting acute kidney injury from multi-organ ARDS shock or circuit-related hemolysis and inflammation [3,5,15]. Arrhythmias occurred in roughly 10% of runs (some due to electrolyte shifts or cannula irritation of the heart), and cardiac arrests requiring cardiopulmonary resuscitation (CPR) occurred in ~5% of patients even while on VV-ECMO (often due to tension pneumothorax, massive hemorrhage, or unforeseen circuit failure) (Figure 4) [15,17].

Barotrauma can also complicate ARDS regardless of ECMO, but interestingly, pneumothorax was reported in ~10% of ECMO patients, sometimes precipitating emergent chest tube placement [17]. This rate is comparable to severe ARDS on mechanical ventilation and may actually decline once on ECMO due to gentler ventilation (Figure 4) [5,15].

Crucially, the risk of serious complications correlates with center experience and protocols. High-volume ECMO centers tend to have lower complication rates; for example, bleeding rates vary widely across studies, partly due to differences in anticoagulation strategies and circuit technology [5,15,17]. A 2019 guideline noted that bleeding complications in literature were "not universally reported or defined," but overall, ECMO carries an 11-fold increased risk of significant bleeding compared to standard care [14,16]. In experienced centers, major hemorrhage has been reported in ~15% of cases, as per ELSO registry data, but in inexperienced hands or during surges (e.g., COVID-19 first wave), higher rates were observed [15,16,17].

The necessity of balancing thrombosis and bleeding risk is a defining challenge of ECMO management, and it underscores why ECMO remains reserved for the most critical cases where benefits outweigh these risks [5,15]. Indeed, candidacy decisions often hinge on whether a patient is robust enough to tolerate possible ECMO-related complications [15,17]. As Supady​ ​​​​​​et al. poignantly stated, ECMO sits between futility and rescue - it should be deployed only when the patient is likely to benefit, given the complexity and "high risk for developing serious complications" inherent to the technique [17].

In summary, while modern ECMO is safer than in the 1970s (due to heparin-bonded circuits, biocompatible surfaces, and improved monitoring), it still carries a considerable morbidity burden [5,15]. Meticulous multidisciplinary care (including perfusionists, intensivists, surgeons, and nurses) is required to identify and manage complications promptly - for example, daily circuit checks for clot, routine imaging for cannula position, aggressive blood product replacement when bleeding, and antibiotic prophylaxis protocols [15]. Many programs implement checklists to ensure early detection of issues like differential hypoxemia, limb ischemia (for femoral cannulation in VA cases), or oxygenator dysfunction [15,17]. The 77.8% overall complication rate reported in one small ARDS series illustrates that virtually no patient on ECMO is complication-free [6]. This reinforces that ECMO is truly a high-stakes intervention, where a successful outcome is defined not just by oxygenation but by averting and surviving these potential pitfalls.

Regional and Population Variability in Outcomes

Outcomes with ECMO in ARDS have shown considerable variability across different regions, centers, and patient populations. In general, high-volume, experienced ECMO centers report better survival than lower-volume centers [15,17]. During the COVID-19 pandemic, this became evident: early international reports from expert centers showed 90-day mortality for COVID-related ARDS on ECMO around 36%-47% (in line with historical ARDS ECMO outcomes) [7,12]. However, as the pandemic progressed and ECMO was utilized in broader settings, some regions noted markedly worse results. By mid-2020, several cohorts (including national audits in Germany and other countries) reported ECMO mortality exceeding 60%-70% [12,16].

This apparent outcome decline was attributed to a combination of factors: patient selection drift (older, sicker patients being cannulated, sometimes after prolonged noninvasive support that caused self-inflicted lung injury), resource strains (ECMO being performed at centers with limited experience due to overwhelming demand), and an increase in secondary infections (from longer ICU stays and immunosuppressive COVID-19 treatments) [7,16,17]. Indeed, an analysis by Hajage et al. found that the survival benefit of ECMO for COVID-19 disappeared by day 90 in an all-comers analysis, but remained present in high-volume centers - suggesting that outcomes were superior when patients were treated at experienced ECMO sites, even during the later pandemic surges [22].

Another large international study of over 17,000 COVID-19 patients treated with ECMO identified care at a low-volume center as an independent risk factor for mortality, along with patient-related factors such as older age and prolonged pre-ECMO mechanical ventilation [16,17]. These findings emphasize that institutional proficiency and real-time case volume can strongly influence ECMO success [15,17].

As a result, there have been regionalization efforts, directing severe ARDS cases to designated ECMO referral centers. For example, in the UK CESAR trial, patients were randomized to transfer to an ECMO center versus stay at local hospitals, and the improved outcome in the transfer group arguably reflected both the use of ECMO and the concentration of care in a specialized center adhering to lung-protective ventilation standards [3,4].

Variability is also seen in different patient subsets. We have already noted the comparatively favorable outcomes in obstetric ARDS (where survival ~70% to 80% on ECMO has been reported) versus geriatric ARDS (where survival drops substantially; octogenarians are rarely offered ECMO due to dismal expected benefit) [6,17]. Immunocompromised patients (e.g., transplant recipients or cancer patients) historically had poor ARDS survival, but recent series suggest ECMO can salvage many of these patients, challenging prior assumptions [5,15]. Some studies indicate immunocompromised status is not an absolute predictor of death on ECMO if the ARDS trigger is reversible [5,15].

Geography and resource availability also play a role. In low- and middle-income countries, ECMO outcomes have been less favorable on average, likely reflecting later initiation and resource constraints [12,17]. For instance, pediatric COVID-19 ECMO mortality was <1% in high-income settings versus up to 10% in low-income settings [12]. Similarly, an adult registry in Latin America during COVID-19 noted higher mortality than ELSO’s global average [16,17].

Single-center experiences illustrate how outcomes can range widely: one tertiary Chinese center (2011-2023) reported only 27.8% survival to hospital discharge among 18 severe ARDS patients treated with ECMO, whereas large multi-center cohorts (e.g., the 2009 H1N1 ECMO series) reported 60%-75% survival [12,19]. Such differences may be owing to case mix (the Chinese series included very prolonged cases and many failures to wean off ECMO), as well as the learning curve of a newer program [19].

The overall historical survival rate for adult VV-ECMO in ARDS is roughly 50%-60% in experienced centers, but this can drop below 30% in suboptimal conditions or exceptionally high-risk groups (e.g., those with chronic comorbidities) [3,5,15]. Notably, regional protocols (such as how long to persist with ECMO before deeming futility, or criteria for lung transplant candidacy) can affect reported outcomes [7,17]. Some centers aggressively pursue lung transplant for ECMO-dependent ARDS patients (which, if successful, turns an otherwise fatal case into a survival), whereas others may withdraw support if lung recovery is deemed unlikely after a few weeks [12,17].

These practice differences mean that *survival* as an endpoint can be influenced by local ethical and resource considerations (e.g., North American and European centers tended to have better COVID-era ECMO outcomes than some Asian reports, possibly because of differing thresholds for offering ECMO and continuing it) [12,16]. Importantly, no robust differences in outcome by gender or ARDS etiology (COVID-19 vs. non-COVID-19, influenza vs. bacterial) have emerged after adjusting for illness severity [3,5]. The key determinants remain illness severity, timing, and center experience rather than ARDS cause per se [5,15,17]. That said, certain causes like COVID-19 introduced unique challenges (e.g., prothrombotic state) as described, and influenza outbreaks can temporarily stress ECMO capacity, leading to triage decisions [7,16].

In summary, the variability in ECMO outcomes underlines that ECMO is not a magic bullet equally effective everywhere - it is highly dependent on patient factors (age, comorbidities, acute severity), system factors (experience, volume, resources), the timing of initiation (with early use, generally within seven days of mechanical ventilation, associated with better outcomes), and even broader contexts such as global health events (early vs. late pandemic) [5,15,16]. This variability provides a cautionary note: when interpreting ECMO studies or planning an ECMO program, one must account for these contextual factors [5,17]. It also speaks to the need for continuous data monitoring, through both global initiatives such as the ELSO Registry and the establishment of regional or national ECMO registries, as well as possibly accreditation of ECMO centers to ensure quality [15,16]. As the use of ECMO grows worldwide, reducing the outcome gap between centers via training, protocols, and tele-ICU support is an active area of focus [5,17].

Clinical implications

ECMO has emerged as a vital rescue therapy for severe ARDS, providing a bridge to recovery in situations of life-threatening refractory respiratory failure. The aggregate evidence from modern trials, meta-analyses, and large cohorts indicates that ECMO can improve survival (approximately 10%-15% absolute risk reduction in 60-90-day mortality) in patients who meet strict criteria for severity [3,4,5]. By diverting blood to an artificial lung, ECMO permits ultraprotective ventilation and ameliorates hypoxemia/hypercapnia, thereby preventing further ventilator-induced lung injury and allowing time for the injured lungs to heal [5,15]. Clinical benefits are seen not only in survival, but in reduced duration of mechanical ventilation and multi-organ support in survivors [3,5]. These outcomes represent a meaningful improvement in the morbidity associated with severe ARDS.

However, the successful application of ECMO requires careful patient selection, timely initiation, and management expertise. The evidence consistently favors using ECMO in younger patients with reversible pathology, initiated within about a week of ARDS onset, and after conventional strategies (including prone positioning and neuromuscular blockade) have failed [4,15,17]. Patients with profound hypoxemia (PaO₂/FiO₂ well below 80) and/or uncompensated hypercapnic acidosis are the ones most likely to benefit [4,5]. In contrast, those with irreversible comorbid conditions or prolonged ventilator courses have less to gain and face higher risks [5,15].

It is also evident that ECMO is not a standalone treatment - outcomes are maximized in specialized centers where a multidisciplinary team can anticipate and manage complications [15,17]. We observed that when ECMO is delivered in experienced centers, outcomes are significantly better than in ad hoc settings [7,12,16]. This argues for regionalizing severe ARDS care and concentrating ECMO cases in high-volume centers of excellence [3,17]. Clinicians referring patients for ECMO should do so early rather than as a last resort during cardiac arrest, since even ECMO has limited utility in moribund patients [5,15]. Once on ECMO, patients require vigilant monitoring for bleeding, thrombosis, and infections, and strategies like daily ultrasound of cannula sites and refined anticoagulation protocols can improve safety [15,16].

The risk-benefit balance of ECMO must be continually assessed for each patient, and a predefined discontinuation plan should ideally be established before initiation of support. Such a plan includes criteria for successful liberation after lung recovery, transition to lung transplantation if feasible, or withdrawal of support in the event of irreversible neurological injury or refractory multiorgan failure [15]. On the other hand, signs of lung recovery (improving compliance, clearing infiltrates) should encourage weaning trials to liberate patients from ECMO as soon as feasible [5,15].

Importantly, new data from the COVID-19 period have reinforced that patient factors (like right ventricular failure or prolonged high-pressure ventilation) critically influence outcomes - severe RV dysfunction on VV-ECMO portends worse survival, and in those cases, teams should consider transitioning to VA-ECMO or adding a pulmonary circulatory support device [7,16].

Moving forward, gaps remain in our knowledge: the field would benefit from further RCTs (e.g., evaluating ECMO in intermediate-severity ARDS, or comparing different ventilator strategies during ECMO), as well as long-term follow-up studies of survivors to assess functional outcomes [3,12]. Moreover, developing standardized protocols to reduce complications (e.g., anticoagulation management algorithms to minimize hemorrhage without incurring thrombosis) is an area of active research [15,17].

Limitations of the evidence and gaps in knowledge

Despite encouraging findings, this review highlights several limitations in the ECMO literature for ARDS. Most conclusions rely on just two modern RCTs - CESAR (*n *= 180) and EOLIA (*n* = 249) - each with design constraints [3,4]. EOLIA was underpowered and allowed 28% crossover to ECMO in the control group, diluting its ability to detect a mortality difference using frequentist analysis [4]. CESAR, while positive, did not mandate ECMO for transferred patients (24% improved with ventilation alone), and 30% of controls did not receive low-tidal-volume ventilation, confounding results [3]. Beyond RCTs, the majority of evidence stems from observational studies and registries, which carry selection bias and center-level confounding despite statistical adjustments [5,15,17]. Variability in center expertise, learning curves, and protocols further complicates interpretation. Moreover, heterogeneity in anticoagulation, transfusion, ventilation practices, and timing of decannulation makes *ECMO *a non-uniform intervention across studies [15,16].

Data on long-term outcomes are also limited. Most studies report in-hospital or 60-90-day outcomes, while few assess quality of life, cognitive function, or long-term pulmonary recovery beyond one year [5,12]. ECMO survivors may have preserved lung function at 6-12 months, but long-term morbidity remains understudied. Economic data were not the primary focus here, but CESAR’s cost-effectiveness analysis estimated approximately £19,000 per Quality-Adjusted Life Year (QALY), reflecting a near-doubling of ICU cost per patient [3]. Additional limitations include publication bias (with centers less likely to publish poor outcomes), lack of consensus on optimal ventilator and anticoagulation strategies during ECMO, and uncertain generalizability to resource-limited settings or community hospitals [5,15,17]. As most studies originate from high-resource ECMO centers, future work should explore program development, cost considerations, and standardized protocols to improve safety and outcomes worldwide.

## Conclusions

The variability in ECMO outcomes underscores that it is not a universally effective intervention; results depend on patient factors (age, comorbidities, acute severity), system factors (experience, volume, resources), timing of initiation (with early use - generally within seven days of mechanical ventilation - associated with better outcomes), and broader contexts, such as global health events (early vs. late pandemic). Delaying initiation until terminal hypoxemia often leads to poor outcomes, whereas timely intervention in eligible patients can prevent this spiral. Overall, the preponderance of evidence supports ECMO as a lifesaving intervention in appropriately selected patients with severe ARDS. When used early, in experienced centers, and for patients without irreversible comorbidities, ECMO improves survival and recovery while maintaining an acceptable safety profile. Nevertheless, it carries substantial risks and resource demands, requiring judicious application and rigorous supportive care. Clinicians should also recognize the limitations of current evidence and the need for ongoing evaluation as technology evolves (e.g., portable devices, safer anticoagulants) and as new causes of ARDS emerge. For now, ECMO stands as the ultimate escalation therapy for ARDS - one that has already saved many lives and, with continued refinements in patient selection and management, is likely to become even more effective in critical care.
